# Divergent functional connectivity during attentional processing in Lewy body dementia and Alzheimer's disease

**DOI:** 10.1016/j.cortex.2017.02.016

**Published:** 2017-07

**Authors:** Xenia Kobeleva, Michael Firbank, Luis Peraza, Peter Gallagher, Alan Thomas, David J. Burn, John O'Brien, John-Paul Taylor

**Affiliations:** a Institute of Neuroscience, Newcastle University, Campus for Ageing and Vitality, Newcastle upon Tyne, UK; b Department of Neurology and Neurophysiology, Medical School Hannover, Hannover, Germany; c Department of Psychiatry, University of Cambridge, Addenbrooke's Hospital, UK; d University Hospital Bonn, Clinic for Neurology, Bonn, Germany

**Keywords:** Dementia with Lewy bodies, Functional magnetic resonance imaging (fMRI), Parkinson's disease dementia, Attention networks, Default mode network, Executive function, Hyperconnectivity, Hypoconnectivity, AD, Alzheimer's disease, ANT, Attention network test, CAF, Clinical assessment of fluctuations, CAMCOG, Cambridge Cognitive Examination, DAN, Dorsal attention network, DMN, Default mode network, EXEC, Executive network, FC, Functional connectivity, LBD, Lewy body dementia, ICA, Independent component analysis, MPFC, Medial prefrontal cortex, MMSE, Mini-Mental State Examination, NPI, Neuropsychiatric Inventory, PCC, Posterior cingulate cortex, ROI, Region of interest, UPDRS, Unified Parkinson's disease rating scale, VAN, Ventral attention network

## Abstract

Attention and executive dysfunction are features of Lewy body dementia (LBD) but their neuroanatomical basis is poorly understood. To investigate underlying dysfunctional attention-executive network (EXEC) interactions, we examined functional connectivity (FC) in 30 patients with LBD, 20 patients with Alzheimer's disease (AD), and 21 healthy controls during an event-related functional magnetic resonance imaging (fMRI) experiment. Participants performed a modified Attention Network Test (ANT), where they were instructed to press a button in response to the majority direction of arrows, which were either all pointing in the same direction or with one pointing in the opposite direction. Network activations during both target conditions and a baseline condition (no target) were derived by (ICA) Independent Component Analysis, and interactions between these networks were examined using the beta series correlations approach.

Our study revealed that FC of ventral and dorsal attention networks DAN was reduced in LBD during all conditions, although most prominently during incongruent trials. These alterations in connectivity might be driven by a failure of engagement of ventral attention networks, and consequent over-reliance on the DAN. In contrast, when comparing AD patients with the other groups, we found hyperconnectivity between the posterior part of the default mode network (DMN) and the DAN in all conditions, particularly during incongruent trials. This might be attributable to either a compensatory effect to overcome DMN dysfunction, or be arising as a result of a disturbed transition of the DMN from rest to task.

Our results demonstrate that dementia syndromes can be characterized both by hyper- and hypoconnectivity of distinct brain networks, depending on the interplay between task demand and available cognitive resources. However these are dependent upon the underlying pathology, which needs to be taken into account when developing specific cognitive therapies for LBD as compared to Alzheimer's.

## 1 Introduction

Lewy body dementia (LBD), which includes both dementia with Lewy bodies and Parkinson's disease dementia, is the second most common cause of neurodegenerative dementia after Alzheimer's disease (AD) (Vann Jones & O'Brien, 2014). In addition to visual hallucinations and parkinsonism, patients frequently experience fluctuating cognition, particularly in the domains of attention and executive function. However the patho-biological underpinnings of these key cognitive symptoms are poorly understood.

It has been long established that neurodegenerative diseases do not simply represent a combination of dysfunctions or lesions of discrete brain areas, but can also be viewed as disconnection syndromes (Morrison et al., 1986). There is a considerable body of evidence showing impaired communication of various brain regions during functional magnetic resonance imaging (fMRI) of blood oxygen level dependent (BOLD) resting state in LBD and AD, particularly affecting attention and executive networks (EXEC) (Franciotti et al., 2013, Peraza et al., 2014; Wang et al., 2006). Another apposite network is the default mode network (DMN) which is active during rest and deactivates during tasks (Binder, 2012, Buckner et al., 2008, Greicius et al., 2003, Shulman et al., 1997), allowing the transfer of neural resources from internal processing to other networks such as attentional networks (Kelly, Uddin, Biswal, Castellanos, & Milham, 2008). In AD, previous studies have reported decreased activity in the DMN during the resting state (Agosta et al., 2012, Dipasquale et al., 2015, Rombouts et al., 2005) whereas in LBD, resting state studies have shown conflicting results, ranging from no change in DMN activity at rest compared to aged controls (Franciotti et al., 2013, Peraza et al., 2014) through to reduced activity (Lowther, O'Brien, Firbank, & Blamire, 2014).

Whilst there are common connectivity patterns during tasks and at rest (Beckmann et al., 2005, Fox et al., 2006), the inter-regional correlations are dynamic and depend on task difficulty, task performance and cognitive state (Cole et al., 2013, Krienen et al., 2014, McIntosh et al., 2003), and thus resting state examinations may only be partially informative. To acquire a fuller understanding of how brain networks are disrupted during attentional and executive dysfunction in LBD, interrogation of brain activity during a task may provide a more complete picture.

In this study of patients with LBD, AD and healthy controls, we analyzed data from a previously reported fMRI dataset (Firbank et al., 2016) acquired by our group where we applied a modified version of the Attention Network Test (ANT), which characterizes dissociable aspects of attention including *alerting*, *orienting* and *conflict* (executive control) (Fan, McCandliss, Sommer, Raz, & Posner, 2002).“ This was performed during fMRI to detect task-related interaction of attentional and EXEC and the DMN. We used independent component analysis (ICA) as a data-driven technique to derive co-activated brain regions throughout the task without the need of an *a priori* hypothesis of a specific network distribution and then compared inter-network connectivity between groups. To explore the relationship between connectivity and attention-executive function, we focussed on the effect of the executive-conflict target stimulus rather than cue elements of the ANT, using beta series correlations; an approach that allows for a separate examination of congruent and incongruent trials (Rissman, Gazzaley, & D'Esposito, 2004).

We hypothesized that we would see a dysfunctional coupling of attentional and EXEC with the DMN in LBD and AD. Specifically, we assumed that the interaction of attention networks would be more impaired in LBD, given generally greater attentional impairment in this patient group, whereas in AD we expected that we would find defective coupling of the DMN with other regions, in accordance with previous task based and resting state studies (Damoiseaux et al., 2012, Dipasquale et al., 2015, Rombouts et al., 2005). We also predicted that disturbances in connectivity would increase during task execution compared to baseline and also in relation to the level of task conflict.

## 2 Methods and participants

### 2.1 Participants

Patients aged over 60 years with mild to moderate dementia (Mini-Mental State Examination – MMSE > 12) were recruited from local old age psychiatry and neurology services. Two experienced senior clinicians applied the revised International Consensus Guidelines for dementia with Lewy bodies (McKeith et al., 2005), Emre criteria for Parkinson's disease with dementia (Emre et al., 2007) and National Institute on Aging (NIA) criteria for AD (McKhann et al., 2011) to independently diagnose probable AD, dementia with Lewy bodies or Parkinson's disease dementia. As our previous imaging findings, in agreement with other studies (McKeith, 2007), did not find notable group differences between dementia with Lewy bodies and Parkinson's disease dementia patients (Firbank et al., 2016) in terms of BOLD activations or cognitive function, we grouped these patients together as an LBD group for our analyses. Severity of parkinsonism was evaluated using the Unified Parkinson's Disease Rating Scale (UPDRS; Fahn, Elton, & UPDRS Development Committee, 1987). We also applied the Neuropsychiatric Inventory (NPI; Cummings et al., 1994), the Mayo Clinic Fluctuation Scale (Ferman et al., 2004) and the clinical assessment of fluctuations (CAF) (Walker et al., 2000). Friends and spouses of the patients in this study and from previous studies participated as healthy control subjects of comparable sex, age and education. This study was approved by the local ethics committee and written consent was obtained from all subjects.

Exclusion criteria for all subjects included contraindications for MR imaging, moderate to severe visual impairment, history of alcohol or substance misuse, significant neurological or psychiatric illnesses (aside from dementia in patient groups), focal brain lesions on brain imaging, evidence of moderate to severe small vessel disease/white matter lesion load, or the presence of other severe or unstable medical illness.

The cognition of all participants was assessed using the Mini Mental State Examination (MMSE; Folstein, Folstein, & McHugh, 1975), Cambridge Cognition Examination (CAMCOG; Roth, Tym, & Mountjoy, 1986); and verbal fluency scores. The Cornell scale for depression in dementia (Alexopoulos, Abrams, Young, & Shamoian, 1988) was used. Visual acuity was measured using Landolt broken rings after correction of refractive errors. Visuospatial function was measured with an angle discrimination task (Mosimann et al., 2004). All LBD patients were scanned in an “on” state with regard to their motor symptoms. Given a possible effect of dopamine-replacement therapy on FC (Tahmasian et al., 2015), we also calculated the levodopa equivalent dosages within the LBD group. All clinical scales and neuropsychological data were compared between groups using independent *t*-tests or ANOVA where appropriate.

### 2.2 Task

**Fig. 1:**
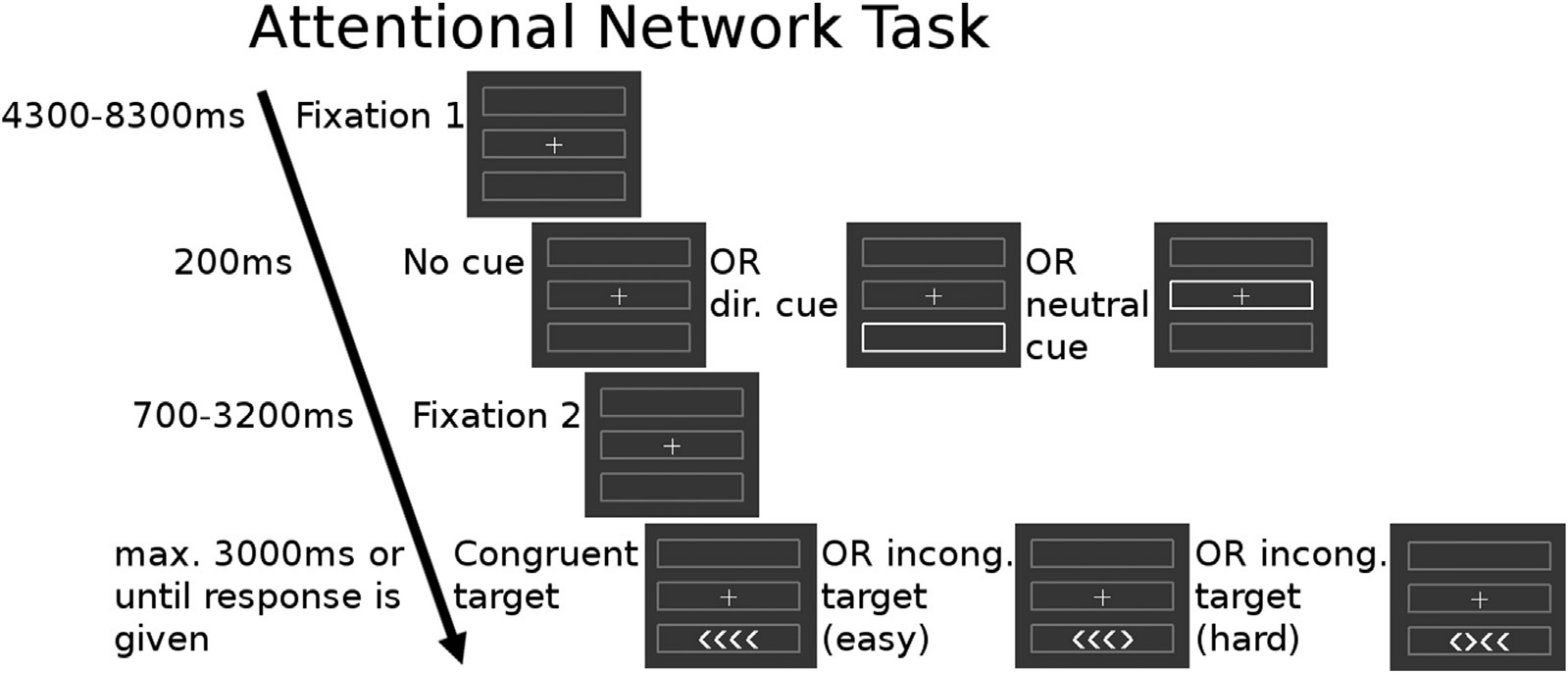
Study design of the ANT.

In one scanning session, we acquired four runs of a modified version of the ANT (Fan et al., 2002, Firbank et al., 2016), which included different grades of conflict (see Fig. 1). The target consisted of four arrowheads, which were either all pointing in the same direction (congruent) or with one of the arrows pointing the opposite direction to the others (incongruent). Subjects had to indicate the direction in which the majority of arrowheads were pointing. Prior to the target, which would be located in the upper or lower box, either no cue, a neutral cue or a directional cue was shown. For each of the four runs the congruent and incongruent target appeared 18 times each, with cue types balanced in each condition. The experiment was programmed using the cogent toolbox (http://www.vislab.ucl.ac.uk/cogent_2000.php) in Matlab (Mathworks, Natick, Massachusetts). The participants were instructed outside of the scanner and also performed at least two trial runs.

### 2.3 fMRI data acquisition

The subjects were scanned in a 3T whole body MR scanner (Achieva scanner; Philips Medical System, the Netherlands) using an 8 channel head coil receiver. We collected a standard structural scan (3D MPRAGE, sagittal acquisition, slice thickness 1.0 mm, in plane resolution 1.0 × 1.0 mm; repetition time (TR) = 8.3 ms; echo time (TE) = 4.6 msec; flip angle = 8°; SENSE factor = 2), and functional scans with a gradient-echo echo planar imaging sequence (TR = 1.92 sec; TE = 40 msec; Field of view 192 × 192 mm^2^ 64 × 64 matrix size, flip angle 90°, 27 slices, slice thickness 3 mm, slice gap 1 mm) with 156 volumes. The participants performed between 4 and 6 runs of the attention task, but only the first four runs were included for data analysis to ensure comparable timings and conditions for the beta series correlations approach.

### 2.4 Data analysis

#### 2.4.1 Preprocessing

The functional data were preprocessed using the FMRI Expert Analysis Tool (FEAT) Version 6.00, part of FMRIB's Software Library (FSL version 5.0, www.fmrib.ox.ac.uk/fsl): Functional scans were motion corrected, followed by removal of non-brain tissue, spatial smoothing with a 6 mm Gaussian kernel, grand-mean intensity normalisation of the entire 4D dataset by a single multiplicative factor and high pass temporal filtering with a cut-off of 100 sec. We also removed non brain tissue of the structural data (threshold gradient *g* = −.2, fractional threshold *f* = .25). Afterwards, we used FSL's linear affine boundary based registration to register the functional images to the structural image. After a linear registration of the structural image to the standard brain space, the functional images were resampled in standard space with voxel size of 4 × 4 × 4 mm. All images were checked manually after these pre-processing steps.

#### 2.4.2 Network region identification

**Fig. 2:**
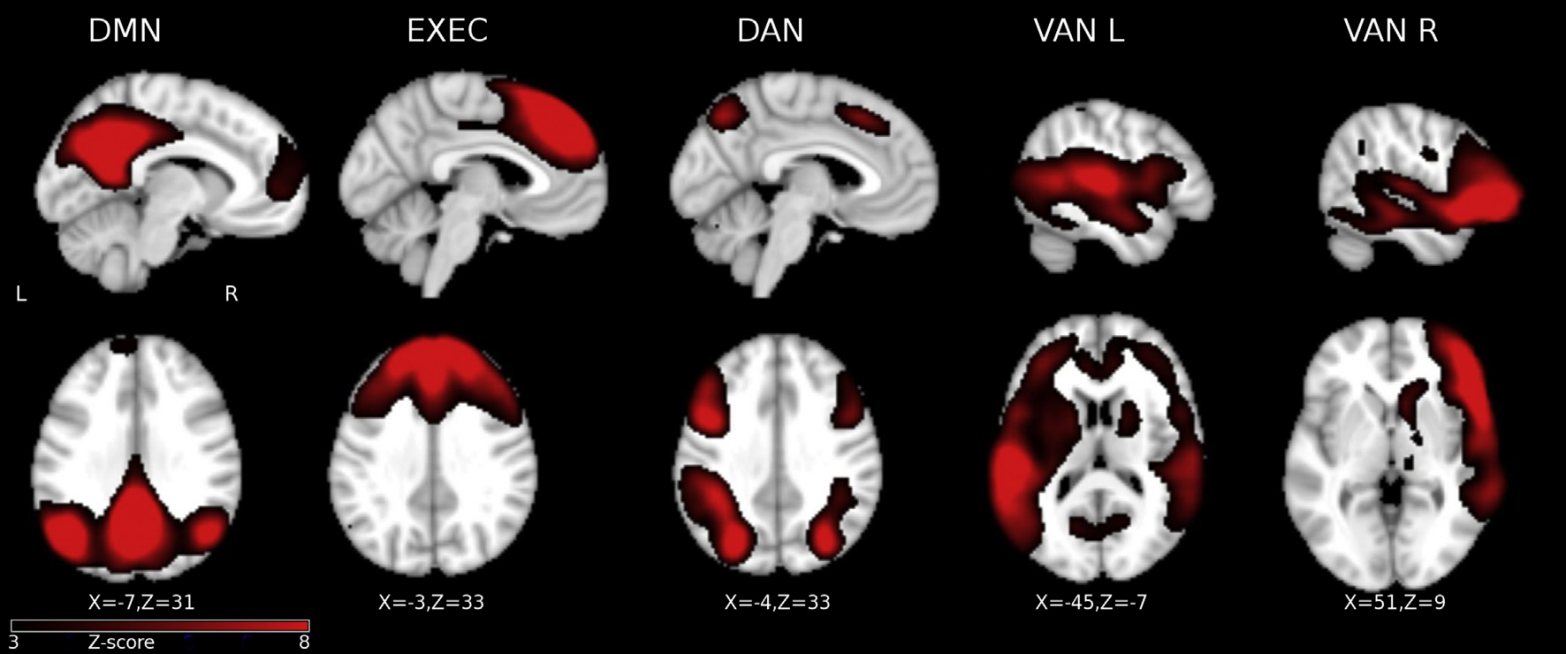
Task-positive and task-negative networks for all groups as revealed by the independent component analysis. Images are shown in radiological convention. DMN, default mode network; EXEC, central executive network; DAN, dorsal attention network; VAN_L, ventral attention network, left; VAN_R, ventral attention network, right.

To extract independent components, fMRI data of all four runs of the ANT were temporally concatenated across all participant groups (healthy controls + AD + LBD) and FSL-MELODIC (http://fsl.fmrib.ox.ac.uk/fsl/fslwiki/MELODIC) was implemented. The number of independent components was estimated automatically (Beckmann & Smith, 2004), resulting in 37 independent components, of which 23 were visually identified as artefactual. We visually selected five components for analysis (see Fig. 2), which were mapped onto the EXEC, DMN and attention networks as based on previous studies (Agosta et al., 2012, Laird et al., 2011, Smith et al., 2009). The remaining components included the insula, sensorimotor networks, frontal pole, occipital lobe and cerebellum, which are not analyzed further in this paper.

To calculate subject-specific time-series, we used FSL's dual regression tool (Beckmann, Mackay, Filippini, & Smith, 2009). The maps of the five selected independent components were regressed against the preprocessed functional data of each subject, resulting in individual time courses for each component.

Given the different putative roles of the anterior and posterior DMN in controls (Uddin, Kelly, Biswal, Castellanos, & Milham, 2009) and AD (Dipasquale et al., 2015, Rombouts et al., 2005, Wang et al., 2007) and to control for spatial overlap between the DMN and the dorsal attention network (DAN), we also extracted two regions of interest (ROI) using a sphere of 10 mm diameter in the anterior and posterior points of maximum activation of the DMN, resulting in posterior cingulate cortex (PCC) and medial prefrontal cortex (MPFC) ROIs.

#### 2.4.3 Beta series activations and correlations

To investigate the effect of the target condition on the inter-network connectivity, we applied the beta series correlation method. This method enables examination of network interactions during each event of an fMRI task and thus comparison of functional connectivity (FC) between different trials (congruent versus incongruent target versus baseline). It is based on the assumption that networks whose beta series are correlated, are also functionally interacting during an event (Rissman et al., 2004). To obtain the beta series, separate GLMs were performed, with the dependent variable being the time-course of each network for each subject, as obtained by the dual regression or from the time courses of the two extracted ROIs (PCC, MPFC). Each individual event was modelled as a separate regressor, with six motion parameters as well as time courses of the cerebrospinal fluid and white matter as nuisance parameters, resulting in parameter estimates (beta estimates) for each individual event per subject and network. Beta estimates for events of interest (baseline, congruent target, incongruent target) were grouped together as beta series. We refer to onset of a congruent target in comparison to the baseline as effect of target, and presentation of the incongruent target in contrast to the congruent target as the effect of conflict.

To examine FC of the networks, the beta series were correlated and then normalized via r-to-z transformation, resulting in covariance matrices for each group, using FSLNETs (http://fsl.fmrib.ox.ac.uk/fsl/fslwiki/FSLNets) and custom Matlab (Mathworks, Natick, Massachusetts) scripts. The covariance matrices were first tested for an overall group difference using a multivariate analysis of variance (Pillai's Trace Methods) and then with pair wise comparisons between groups using two-sample *t*-tests for group contrasts (controls *vs* LBD, controls *vs* AD). To check if the observed effects were disease-specific, we also performed a comparison between AD and LBD. All resulting *z*- and *p*-values were FDR-corrected for multiple comparisons using the Benjamini Hochberg procedure unless stated otherwise. In case of significant group differences for the covariance matrices, we explored whether these were correlated with clinical scores related to attentional and cognitive dysfunction (CAMCOG, MMSE, CAF total score and MAYO fluctuation subscale) and levodopa equivalent doses (within the LBD group) correcting the resulting *p*-values with the Bonferroni procedure.

We also calculated mean activations for each condition of interest (baseline, congruent target, incongruent target) and group, and submitted them to a 3 (condition) × 3 (group) repeated-measures ANOVA for each network. In case of violation of sphericity, a Greenhouse-Geisser correction was performed. Post-hoc *t*-tests (paired samples *t*-tests for condition and independent samples *t*-tests for group) were performed and then corrected for multiple comparisons using the Bonferroni procedure [Results in supplementary material, Fig. S1].

## 3 Results

### 3.1 Demographic and clinical data

Table 1 summarizes the demographic and clinical data of the controls, LBD and AD group. As expected, the LBD group had significantly higher UPDRS and CAF scores, MAYO fluctuations subscale and total score, and NPI hallucinations subscale and NPI total score than control or AD groups.

**Table 1:** Demographical data of controls, AD and LBD subjects.

	Controls		Alzheimer's disease		LBD		AD versus LBD
	Mean N = 21	SD	Mean N = 20	SD	Mean N = 30	SD	*p*-value
Age (in years)	76,4	5,4	75,0	8,4	74,7	6,5	.868
Gender (M:F)	15:6		17:3		26:4		.590
CAMCOG total score	96,5	3,6	72,2	11,4	76,7	12,9	.208
CAMCOG executive score	22,7	2,3	15,2	4,4	13,1	4,2	.603
MMSE	29,0	0,9	22,4	3,3	23,3	3,9	.357
Verbal fluency (FAS)	41,7	15,5	31,7	16,1	20,1	12,2	.087
**UPDRS**	1,4	1,8	2,0	1,8	19,3	8,2	<**.001**
**Cornell**	0,5	0,9	1,0	1,1	3,0	2,2	**.002**
**CAF total score**			0,6	1,5	5,1	4,1	<**.001**
**MAYO Clinic Fluctuation Scale, fluctuation subscore**			0,9	1,0	2,4	1,4	<**.001**
MAYO Clinic Fluctuation Scale, cognitive subscore			1,9	2,0	2,8	1,9	.143
**MAYO Clinic Fluctuation Scale, total score**			8,6	4,6	13,9	6,2	**.002**
**NPI hallucinations subscore**			3,5	1,7	3,4	2,1	<**.001**
**NPI total score**			0,0	0,0	1,9	2,4	**.006**
Dopaminergic medication (%)			6,8	7,1	14,0	10,1	
Cholinergic medication (%)			0,0		73,3		

Bold letters mean that the p-value is less than p > 0.05.

CAF, Clinical Assessment of Fluctuations; CAMCOG, Cambridge Cognitive Assessment; NPI, Neuropsychiatric Inventory; MMSE, Mini Mental State Examination; UPDRS, Unified Parkinson Disease Rating Scale.

### 3.2 Behavioral data

The AD and LBD groups had higher error rates and longer reaction times than the control group, but there was no difference in error rates between AD and LBD. The reaction times were significantly longer in LBD than in AD both during the congruent and incongruent condition (see Table 2).

**Table 2:** Reaction times (RT) and error rates for all three groups.

	Controls		AD		LBD		AD versus LBD
	Mean	SD	Mean	SD	Mean	SD	*p*-value
Congruent target: error rate (%)	1.32	1.55	4.24	5.73	4.86	5.28	.694
Incongruent target: error rate (%)	1.72	1.31	10.76	10.78	16.34	12.74	.114
**Congruent target: RT (msec)**	898.33	108.32	1064.38	193.49	1296.61	250.21	**.001**
**Congruent target: RT (msec)**	1233.91	235.27	1543.38	310.35	1850.03	419.07	**.007**

Bold letters mean that the *p*-value is less than *p* > 0.05.

### 3.3 Beta series correlations

#### 3.3.1 Control group

**Fig. 3:**
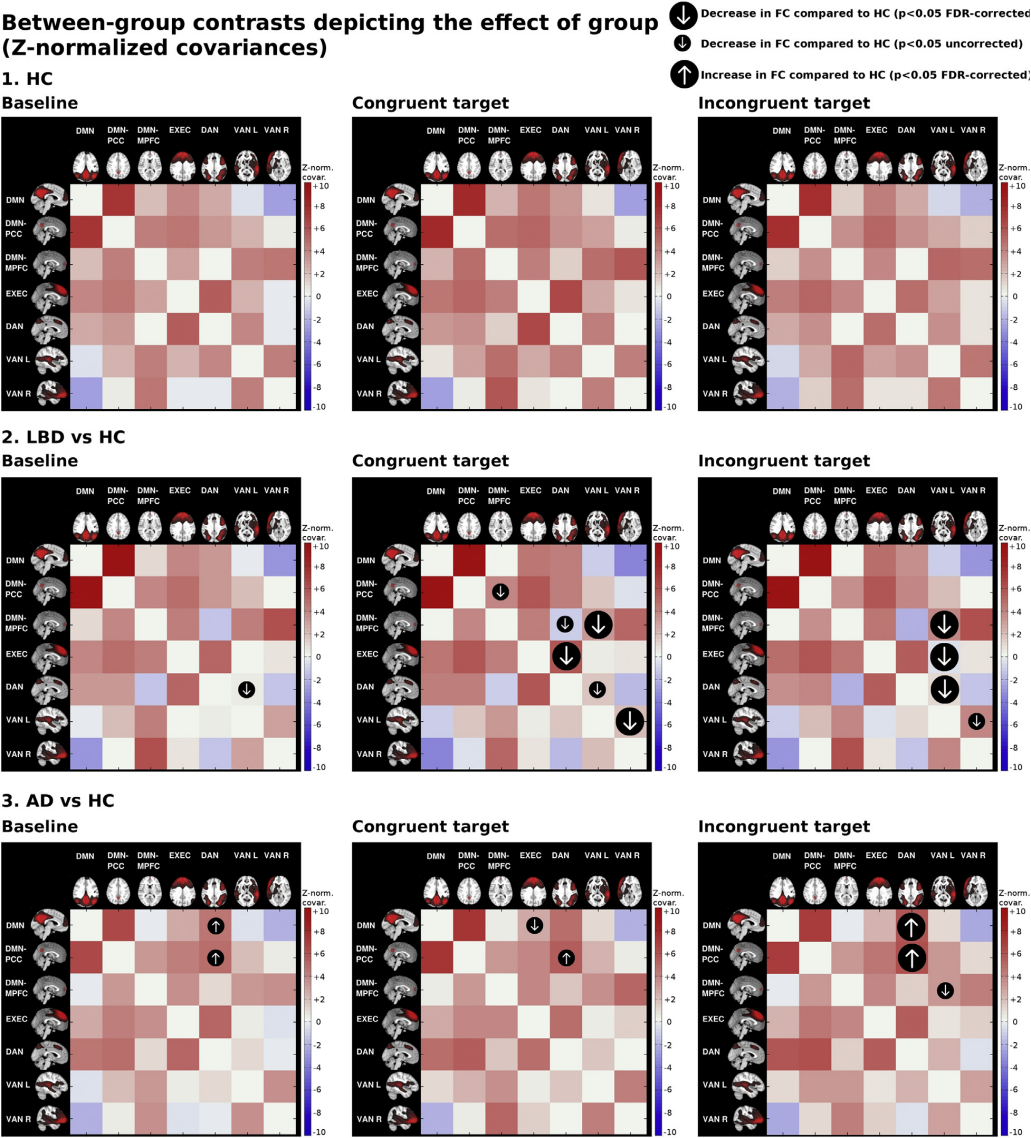
Between-group contrasts in functional connectivity (FC). Group-level covariance matrices displaying the Z-normalised covariance coefficients of the beta series for each network during different trial conditions (baseline, congruent target, incongruent target). The group maps are overlaid with arrows depicting the significant group contrasts (small arrow: *p* < .05 uncorrected, large arrow: *p* < .05 FDR-corrected). AD, AD; DMN, default mode network; EXEC, central executive network; DAN, dorsal attention network; HC, healthy controls; IC, independent component; LBD, Lewy body dementia; VAN_L, ventral attention network, left; VAN_R, ventral attention network, right.

Results from the control group are shown in Fig. 3 and Table S1. In all three conditions, the DAN was positively correlated with the PCC. There was also a positive correlation of the anterior and posterior DMN with the EXEC. The EXEC itself was positively coupled with the DAN and we also found a positive correlation of the MPFC with the ventral attention networks. The left VAN was positively coupled with the right VAN. Presentation of either the congruent or incongruent target (compared to baseline) led to a stronger within network coupling of the DMN as a whole with the PCC. There was also stronger coupling between the MPFC and the right VAN and the EXEC correlated more strongly with the DAN during target presentation. When comparing changes related to the incongruent target (*vs* congruent), connectivity was primarily altered in two regions; firstly, there was less coupling between the left VAN and DMN and secondly, the connectivity between the EXEC and DAN decreased.

#### 3.3.2 Group comparisons

The multivariate analysis of variance was significant for a group effect for all three conditions (baseline: *p* = .005, congruent: *p* = .009, incongruent: *p* = .007). Significant group contrasts as derived from the *t*-tests were mapped onto each group's connectivity matrices, both with un-corrected and corrected *p*-values are shown in Fig. 3. The complete z-scores and *p*-values for all groups and conditions can be found in Tables S1 and S2 of the supplementary material.

##### 3.3.2.1 LBD versus controls

We found no increase in FC in the LBD group when contrasted against the control group. In the baseline condition, only the FC between the left VAN and DAN was decreased (*p* = .006 uncorrected, *p* = .126 FDR-corrected) between LBD and controls. In contrast, during the congruent condition we found a significant decrease in connectivity between the DAN and several regions in LBD (DAN–MPFC: *p* = .039 uncorrected; DAN–EXEC: *p* = .038 FDR-corrected) and the left VAN showed decreased FC to a number of other regions as well (left VAN–MPFC: *p* = .017 FDR-corrected, left VAN–DAN: *p* = .035 uncorrected, left VAN–right VAN: *p* = .017 FDR-corrected).

The incongruent condition led to a stronger decrease in FC of the left VAN in LBD compared to controls with other examined regions (left VAN–MPFC: *p* = .020 FDR-corrected, left VAN–EXEC: *p* = .048 FDR-corrected, left VAN–DAN: *p* = .048 FDR-corrected, left VAN–right VAN: *p* = .040 uncorrected) than the congruent condition.

##### 3.3.2.2 AD versus controls

The baseline condition led to an increase in connectivity between the DMN and DAN in the AD group compared to controls, specifically between the PCC and DAN. In the congruent condition, the increase of coupling between PCC and DAN remained in the AD group compared to controls. We also found a decrease in connectivity in AD versus controls between EXEC and DMN. Apart from the increase of FC between DMN and DAN in AD versus controls, which were already present in the congruent condition, we additionally found a decrease in FC between MPFC and left VAN in AD versus controls in the incongruent condition.

##### 3.3.2.3 AD versus LBD

No increase in FC in LBD compared to AD could be found in any condition, but we found increases in FC in AD compared to LBD (see Table S2 in the supplementary material); in all three conditions there was an increase in FC between DAN and DMN in AD versus LBD, especially the PCC, similar to what we observed in AD versus controls. We also found an increase in coupling of left VAN and right VAN in AD versus LBD during the baseline and congruent condition. During the congruent and incongruent condition, we found a decrease in FC between PCC and left VAN in LBD compared to AD and additionally between MPFC and left VAN during the congruent condition only.

##### 3.3.2.4 Correlations with clinical variables

Correlations with the examined clinical variables and network activity did not reach significance in any group after Bonferroni-correction. There were no significant correlations of the FC values with the levodopa equivalent dosages in the LBD group.

## 4 Discussion

We found diverging inter-network connectivity patterns in both diseases; the connectivity of attention networks with each other and with EXEC was lower in LBD, whereas we observed a higher connectivity between the DMN and dorsal attention and EXEC in AD.

### 4.1 Hypoconnectivity of attention networks in LBD

Only a few studies have examined correlations between brain region activations in LBD, revealing decreased coupling between frontoparietal regions during rest (Franciotti et al., 2013, Peraza et al., 2014) and no prior studies have focussed on task related FC changes using fMRI. In our study, we observed that target presentation during an executive task led to a decreased connectivity of the DAN and VAN with each other as well as with frontal networks such as the EXEC and MPFC in the LBD group. Presentation of the congruent target involved both disconnection of the DAN and VAN in LBD, whereas conflict processing as induced by the incongruent target intensified the decreased coupling of the VAN.

Accurate attentional functioning depends highly on a bilateral and dynamic communication between the DAN and VAN (Daitch et al., 2013, Parks and Madden, 2013, Wen et al., 2012). Depending on goals and target expectations, the DAN facilitates top-down attention processing by suppressing the VAN to exclude irrelevant bottom-up information (Bressler et al., 2008, Corbetta and Shulman, 2002, Stokes et al., 2009). Conversely, the VAN as a stimulus-driven network sends bottom-up signals to the DAN and other higher cortical areas (Corbetta, Patel, & Shulman, 2008). Although the VAN is usually right-dominant (Vossel, Geng, & Fink, 2014), we found a co-activation of the left VAN, which might be due to task design and contrast selection as stated in other studies (DiQuattro and Geng, 2011, Doricchi et al., 2010).

We suggest that the reduced coupling between the DAN and the VAN in LBD may therefore reflect the disturbed attention processing which typifies this type of dementia (Bradshaw, Saling, Anderson, Hopwood, & Brodtmann, 2006; Ferman et al., 2006). The switching and interaction between the DAN and VAN appears to be disconnected in DLB during the presentation of an external stimulus, which normally requires an integration of both networks for intact attention function. We speculate that, given the normal activation of the DAN but reduced activation of the VAN (Fig. S1), the disconnection might be a regulatory process to decrease corrupted bottom-up information transfer from the VAN to the DAN, leading to an over-reliance on the DAN in LBD so that external stimuli such as the target are processed less efficiently.

There was also evidence of an involvement of the anterior DMN in the disrupted FC of attention networks in LBD. Given there is an excessive deactivation of the DMN in LBD (Firbank et al., 2016), it seems that although the DMN was deactivated on task onset, there was inefficient communication and possibly less effective resource allocation between DMN and attention networks, which might be mediated by dysfunction in the VAN rather than DMN.

We also detected a decreased coupling of the DAN and VAN with the EXEC. The EXEC network is implicated in functions such as conflict processing, maintenance of an attention set and activation of a correct behavioral response (Banich et al., 2000, Coderre and van Heuven, 2013, Dosenbach et al., 2008, Egner and Hirsch, 2005, Milham et al., 2003). This may imply in LBD that a defective interaction between the DAN and VAN and the EXEC could indicate a disturbed information transfer from the attention networks to the EXEC as well as a reduced orchestration of attention networks by an impaired EXEC, which in itself may be a contributor to aberrant conflict decisions and less efficient maintenance of an attention set.

Bringing these findings together, we observed a reduced interaction of bottom-up networks such as the VAN with the DAN and EXEC in response to an external task stimulus as well as less interconnection of the attention networks with the EXEC causing possibly less regulation of these networks by the EXEC, which we would argue leads to an overreliance on internal expectations of the external world by the DAN. This might result in less flexible adaptation to task demands and less efficient switching between internal and external cognitive modes, as evidenced by the reduced connectivity of DMN and VAN and excessive DMN deactivation in LBD (Firbank et al., 2016).

Contrarily, there is good evidence to suggest impairment of the DAN in the context of internally generated hallucinations or the perception of ambiguous, delusional stimuli (Collerton et al., 2005, Frith et al., 2000, Heitz et al., 2015, Muller et al., 2014, Shine et al., 2014). Therefore whilst there may be common pathologies underlying attentional dysfunction and hallucinations (Bronnick et al., 2011, Cagnin et al., 2013, Uchiyama et al., 2012), there may be diverging network deficits which are situationally dependent upon internal versus external expectations.

### 4.2 Hyperconnectivity of the DMN and DAN in AD

In AD, connectivity changes were mainly related to the DMN with an emphasis on the posterior aspect of this network. We found a decreased connectivity between DMN and EXEC during the congruent condition and higher FC between DMN and DAN, especially the PCC and the DAN, in all conditions, which increased in relation to the conflict level.

Hyperconnectivity in AD has been observed during many resting state studies, especially in the anterior regions, whereas posterior regions have been associated with hypoconnectivity (Balthazar et al., 2014, Damoiseaux et al., 2012, Jones et al., 2011). In contrast, our task-related data point toward hyperconnectivity of the posterior DMN, indicating a task-dependent effect. Functional hyperconnectivity depends on the degree of task difficulty and also availability of cognitive resources (Catani and ffytche, 2005, Hillary et al., 2014, McLaren et al., 2014): increasing difficulty requires more correlation or anticorrelation depending on the type of interaction, but there is a limit which is defined by the neural capacities of each individual. Given our AD group was relatively mild in terms of cognitive impairment, one explanation for the hyperconnectivity we observed is that it is a compensatory response, as suggested by previous resting state studies (Bai et al., 2009, Damoiseaux et al., 2012, Li et al., 2012). However the observed hyperconnectivity could also be a sign of a failure to decouple the DMN from competing attentional and EXEC during goal-directed behavior (Sharp et al., 2011) and a disturbance of transition between networks from DMN activation during rest to DMN deactivation during task, which is needed to shift neural resources from internally-focussed to externally-oriented processes (Anticevic et al., 2012).

### 4.3 Limitations and future directions

This study examined which network interactions are altered in LBD and AD, but being a correlative approach it cannot provide information on directionality or causality. However our findings may provide a basis for effective connectivity analyses (Friston, 2011), which might shed light on whether the attention and executive dysfunction in LBD is a function of aberrant top-down or bottom-up processing or both. Another limitation is the observed changes between the groups may have been partly driven by reaction time differences between groups (Weissman, Roberts, Visscher, & Woldorff, 2006). We also failed to observe significant associations between clinical and cognitive correlates and FC despite clear group differences. Part of this may reflect our conservative approach with correlations by use of Bonferroni correction to avoid false positives and also the fact that our patients were relatively mild and the range of impairments was not wide. Future studies examining *a priori* the networks demarcated as abnormal in our study will allow for a more nuanced clarification of whether the severity of cognitive impairment clinically map onto these specific networks.

## 5 Conclusions

FC of distant brain regions is a dynamic process and depends on task demands and required neural resources (Krienen et al., 2014, Rissman et al., 2004). In AD attention and executive functioning was dominated by hyperconnectivity of the DMN with the DAN, which may be a sign of either compensation or a failure to decouple the DMN from attentional and EXEC during the transition from rest to task. A more distributed hypoconnectivity of DAN and VAN with each other, as well as with frontal regions was seen in LBD, which is possibly mediated by an impaired bottom-up ventral attentional networks and an over-reliance on top-down dorsal attentional networks and also a dysfunction of the EXEC. Our findings underline that FC analyses are powerful tools to detect early disruptions as well as compensatory processes within functional domains. Furthermore our data also indicate that, while there is general consensus that structural disconnection is a common pathological feature in dementia, dementia syndromes can be regarded as dynamic disorders of both hyper- and hypoconnectivity (Catani and ffytche, 2005, Hillary et al., 2015).

## Conflict of interest

All authors have no conflict of interest in regard to this publication.

## References

[bib1] Agosta F., Pievani M., Geroldi C., Copetti M., Frisoni G.B., Filippi M. (2012). Resting state fMRI in Alzheimer's disease: Beyond the default mode network. Neurobiology of Aging.

[bib2] Alexopoulos G.S., Abrams R.C., Young R.C., Shamoian C.A. (1988). Cornell scale for depression in dementia. Biological Psychiatry.

[bib3] Anticevic A., Cole M.W., Murray J.D., Corlett P.R., Wang X.-J., Krystal J.H. (2012). The role of default network deactivation in cognition and disease. Trends in Cognitive Sciences.

[bib4] Bai F., Watson D.R., Yu H., Shi Y., Yuan Y., Zhang Z. (2009). Abnormal resting-state functional connectivity of posterior cingulate cortex in amnestic type mild cognitive impairment. Brain Research.

[bib5] Balthazar M.L.F., Pereira F.R.S., Lopes T.M., da Silva E.L., Coan A.C., Campos B.M. (2014). Neuropsychiatric symptoms in Alzheimer's disease are related to functional connectivity alterations in the salience network. Human Brain Mapping.

[bib6] Banich M.T., Milham M.P., Atchley R.a., Cohen N.J., Webb A., Wszalek T. (2000). Prefrontal regions play a predominant role in imposing an attentional “set”: Evidence from fMRI. Cognitive Brain Research.

[bib7] Beckmann C.F., DeLuca M., Devlin J.T., Smith S.M. (2005). Investigations into resting-state connectivity using independent component analysis. Philosophical Transactions of the Royal Society of London. Series B, Biological Sciences.

[bib8] Beckmann, Mackay, Filippini, Smith (2009). Group comparison of resting-state FMRI data using multi-subject ICA and dual regression. NeuroImage.

[bib9] Beckmann C.F., Smith S.M. (2004). Probabilistic independent component analysis for functional magnetic resonance imaging. IEEE Transactions on Medical Imaging.

[bib10] Binder J.R. (2012). Task-induced deactivation and the “resting” state. NeuroImage.

[bib11] Bradshaw J.M., Saling M., Anderson V., Hopwood M., Brodtmann A. (2006). Higher cortical deficits influence attentional processing in dementia with Lewy bodies, relative to patients with dementia of the Alzheimer's type and controls. Journal of Neurology, Neurosurgery, and Psychiatry.

[bib12] Bressler S.L., Tang W., Sylvester C.M., Shulman G.L., Corbetta M. (2008). Top-down control of human visual cortex by frontal and parietal cortex in anticipatory visual spatial attention. The Journal of Neuroscience: the Official Journal of the Society for Neuroscience.

[bib13] Bronnick K., Emre M., Tekin S., Haugen S.B., Aarsland D. (2011). Cognitive correlates of visual hallucinations in dementia associated with Parkinson's disease. Movement Disorders.

[bib14] Buckner R.L., Andrews-Hanna J.R., Schacter D.L. (2008). The brain's default network: Anatomy, function, and relevance to disease. Annals of the New York Academy of Sciences.

[bib15] Cagnin A., Gnoato F., Jelcic N., Favaretto S., Zarantonello G., Ermani M. (2013). Clinical and cognitive correlates of visual hallucinations in dementia with Lewy bodies. Journal of Neurology, Neurosurgery, and Psychiatry.

[bib16] Catani M., ffytche D.H. (2005). The rises and falls of disconnection syndromes. Brain: a Journal of Neurology.

[bib17] Coderre E.L., van Heuven W.J. (2013). Modulations of the executive control network by stimulus onset asynchrony in a Stroop task. BMC Neuroscience.

[bib18] Cole M.W., Reynolds J.R., Power J.D., Repovs G., Anticevic A., Braver T.S. (2013). Multi-task connectivity reveals flexible hubs for adaptive task control. Nature Neuroscience.

[bib19] Collerton D., Perry E., McKeith I. (2005). Why people see things that are not there: A novel perception and attention deficit model for recurrent complex visual hallucinations. The Behavioral and Brain Sciences.

[bib20] Corbetta M., Patel G., Shulman G.L. (2008). The reorienting system of the human brain: From environment to theory of mind. Neuron.

[bib21] Corbetta M., Shulman G.L. (2002). Control of goal-directed and stimulus-driven attention in the brain. Nature Reviews. Neuroscience.

[bib22] Cummings J.L., Mega M., Gray K., Rosenberg-Thompson S., Carusi D.A., Gornbein J. (1994). The neuropsychiatric inventory: Comprehensive assessment of psychopathology in dementia. Neurology.

[bib23] Daitch A.L., Sharma M., Roland J.L., Astafiev S.V., Bundy D.T., Gaona C.M. (2013). Frequency-specific mechanism links human brain networks for spatial attention. Proceedings of the National Academy of Sciences of the United States of America.

[bib24] Damoiseaux J.S., Prater K.E., Miller B.L., Greicius M.D. (2012). Functional connectivity tracks clinical deterioration in Alzheimer's disease. Neurobiology of Aging.

[bib25] Dipasquale O., Griffanti L., Clerici M., Nemni R., Baselli G., Baglio F. (2015). High-dimensional ICA analysis detects within-network functional connectivity damage of default-mode and sensory-motor networks in Alzheimer's disease. Frontiers in Human Neuroscience.

[bib26] DiQuattro N.E., Geng J.J. (2011). Contextual knowledge configures attentional control networks. Journal of Neuroscience.

[bib27] Doricchi F., MacCi E., Silvetti M., MacAluso E. (2010). Neural correlates of the spatial and expectancy components of endogenous and stimulus-driven orienting of attention in the posner task. Cerebral Cortex.

[bib28] Dosenbach N.U.F., Fair D.A., Cohen A.L., Schlaggar B.L., Petersen S.E. (2008). A dual-networks architecture of top-down control. Trends in Cognitive Sciences.

[bib29] Egner T., Hirsch J. (2005). The neural correlates and functional integration of cognitive control in a Stroop task. NeuroImage.

[bib30] Emre M., Aarsland D., Brown R., Burn D.J., Duyckaerts C., Mizuno Y. (2007). Clinical diagnostic criteria for dementia associated with Parkinson's disease. Movement Disorders: official Journal of the Movement Disorder Society.

[bib31] Fahn S., Elton R.L., Fahn S., Marsden C.D., Calne D.B., Goldstein M., UPDRS Development Committee, M. of the (1987). Recent developments in Parkinson's disease.

[bib32] Fan J., McCandliss B.D., Sommer T., Raz A., Posner M.I. (2002). Testing the efficiency and independence of attentional networks. Journal of Cognitive Neuroscience.

[bib33] Ferman T.J., Smith G.E., Boeve B.F., Graff-Radford N.R., Lucas J.A., Knopman D.S. (2006). Neuropsychological differentiation of dementia with Lewy bodies from normal aging and Alzheimer's disease. The Clinical Neuropsychologist.

[bib34] Ferman T.J., Smith G.E., Boeve B.F., Ivnik R.J., Petersen R.C., Knopman D. (2004). DLB fluctuations: Specific features that reliably differentiate DLB from AD and normal aging. Neurology.

[bib35] Firbank M., Kobeleva X., Cherry G., Killen A., Gallagher P., Burn D.J. (2016). Neural correlates of attention-executive dysfunction in lewy body dementia and Alzheimer's disease. Human Brain Mapping.

[bib36] Folstein M.F., Folstein S.E., McHugh P.R. (1975). “Mini-mental state”. A practical method for grading the cognitive state of patients for the clinician. Journal of Psychiatric Research.

[bib37] Fox M.D., Corbetta M., Snyder A.Z., Vincent J.L., Raichle M.E. (2006). Spontaneous neuronal activity distinguishes human dorsal and ventral attention systems. Proceedings of the National Academy of Sciences of the United States of America.

[bib38] Franciotti R., Falasca N.W., Bonanni L., Anzellotti F., Maruotti V., Comani S. (2013). Default network is not hypoactive in dementia with fluctuating cognition: An Alzheimer disease/dementia with Lewy bodies comparison. Neurobiology of Aging.

[bib39] Friston K.J. (2011). Functional and effective connectivity: A review. Brain Connectivity.

[bib40] Frith C.D., Blakemore S.J., Wolpert D.M. (2000). Explaining the symptoms of schizophrenia: Abnormalities in the awareness of action. Brain Research Reviews.

[bib41] Greicius M.D., Krasnow B., Reiss A.L., Menon V. (2003). Functional connectivity in the resting brain: A network analysis of the default mode hypothesis. Proceedings of the National Academy of Sciences of the United States of America.

[bib42] Heitz C., Noblet V., Cretin B., Philippi N., Kremer L., Stackfleth M. (2015). Neural correlates of visual hallucinations in dementia with Lewy bodies. rthritiAlzheimer's Research & Therapy.

[bib43] Hillary F.G., Roman C.A., Venkatesan U., Rajtmajer S.M., Bajo R., Castellanos N.D. (2014). Hyperconnectivity is a fundamental response to neurological disruption. Neuropsychology.

[bib44] Hillary F.G., Roman C.A., Venkatesan U., Rajtmajer S.M., Bajo R., Castellanos N.D. (2015). Hyperconnectivity is a fundamental response to neurological disruption. Neuropsychology.

[bib45] Jones D.T., MacHulda M.M., Vemuri P., McDade E.M., Zeng G., Senjem M.L. (2011). Age-related changes in the default mode network are more advanced in Alzheimer disease. Neurology.

[bib46] Kelly a.M.C., Uddin L.Q., Biswal B.B., Castellanos F.X., Milham M.P. (2008). Competition between functional brain networks mediates behavioral variability. NeuroImage.

[bib47] Krienen F.M., Yeo B.T.T., Buckner R.L., Buckner R.L. (2014). Reconfigurable task-dependent functional coupling modes cluster around a core functional architecture.

[bib48] Laird A.R., Fox P.M., Eickhoff S.B., Turner J.A., Ray K.L., McKay D.R. (2011). Behavioral interpretations of intrinsic connectivity networks. Journal of Cognitive Neuroscience.

[bib49] Li B., Wang X., Yao S., Hu D., Friston K. (2012). Task-dependent modulation of effective connectivity within the default mode network. Frontiers in Psychology.

[bib50] Lowther E.R., O'Brien J.T., Firbank M.J., Blamire A.M. (2014). Lewy body compared with Alzheimer dementia is associated with decreased functional connectivity in resting state networks. Psychiatry Research - Neuroimaging.

[bib51] McIntosh A.R., Rajah M.N., Lobaugh N.J. (2003). Functional connectivity of the medial temporal lobe relates to learning and awareness. The Journal of Neuroscience: the Official Journal of the Society for Neuroscience.

[bib52] McKeith I. (2007). Dementia with Lewy bodies and Parkinson's disease with dementia: Where two worlds collide. Practical Neurology.

[bib53] McKeith I.G., Dickson D.W., Lowe J., Emre M., O'Brien J.T., Feldman H. (2005). Diagnosis and management of dementia with Lewy bodies: Third report of the DLB consortium. Neurology.

[bib54] McKhann G.M., Knopman D.S., Chertkow H., Hyman B.T., Jack C.R., Kawas C.H. (2011). The diagnosis of dementia due to Alzheimer's disease: Recommendations from the National Institute on Aging-Alzheimer's Association workgroups on diagnostic guidelines for Alzheimer's disease. Alzheimer's and Dementia.

[bib55] McLaren D.G., Sperling R.a., Atri A. (2014). Flexible modulation of network connectivity related to cognition in alzheimer's disease. NeuroImage.

[bib56] Milham M.P., Banich M.T., Claus E.D., Cohen N.J. (2003). Practice-related effects demonstrate complementary roles of anterior cingulate and prefrontal cortices in attentional control. NeuroImage.

[bib57] Morrison J.H., Scherr S., Lewis D.A., Campbell M.J., Bloom F.E., Rogers J., Scheibel A.B., Wechsler A.F., Brazier M.A.B. (1986). The laminar and regional distribution of neocortical somatostatin and neuritic plaques: Implications for Alzheimer's disease as a global neocortical disconnection syndrome. The biological substrates of Alzheimer's disease.

[bib58] Mosimann U.P., Mather G., Wesnes K.A., O'Brien J.T., Burn D.J., McKeith I.G. (2004). Visual perception in Parkinson disease dementia and dementia with Lewy bodies. Neurology.

[bib59] Muller A.J., Shine J.M., Halliday G.M., Lewis S.J.G. (2014). Visual hallucinations in Parkinson's disease: Theoretical models. Movement Disorders.

[bib60] Parks E.L., Madden D.J. (2013). Brain connectivity and visual attention. Brain Connectivity.

[bib61] Peraza L.R., Kaiser M., Firbank M., Graziadio S., Bonanni L., Onofrj M. (2014). FMRI resting state networks and their association with cognitive fluctuations in dementia with Lewy bodies. NeuroImage: clinical.

[bib62] Rissman J., Gazzaley A., D'Esposito M. (2004). Measuring functional connectivity during distinct stages of a cognitive task. NeuroImage.

[bib63] Rombouts S.A.R.B., Barkhof F., Goekoop R., Stam C.J., Scheltens P. (2005). Altered resting state networks in mild cognitive impairment and mild Alzheimer's disease: An fMRI study. Human Brain Mapping.

[bib64] Roth M., Tym E., Mountjoy C.Q. (1986). CAMDEX. A standardised instrument for the diagnosis of mental disorder in the elderly with special reference to the early detection of dementia. British Journal of Psychiatry.

[bib65] Sharp D.J., Beckmann C.F., Greenwood R., Kinnunen K.M., Bonnelle V., De Boissezon X. (2011). Default mode network functional and structural connectivity after traumatic brain injury. Brain.

[bib66] Shine J.M., Halliday G.M., Gilat M., Matar E., Bolitho S.J., Carlos M. (2014). The role of dysfunctional attentional control networks in visual misperceptions in Parkinson's disease. Human Brain Mapping.

[bib67] Shulman G.L., Fiez J.a., Corbetta M., Buckner R.L., Miezin F.M., Raichle M.E. (1997). Common blood flow changes across visual tasks: II. Decreases in cerebral cortex. Journal of Cognitive Neuroscience.

[bib68] Smith S.M., Fox P.T., Miller K.L., Glahn D.C., Fox P.M., Mackay C.E. (2009). Correspondence of the brain's functional architecture during activation and rest. Proceedings of the National Academy of Sciences of the United States of America.

[bib69] Stokes M., Thompson R., Nobre A.C., Duncan J. (2009). Shape-specific preparatory activity mediates attention to targets in human visual cortex. Proceedings of the National Academy of Sciences of the United States of America.

[bib70] Tahmasian M., Bettray L.M., van Eimeren T., Drzezga A., Timmermann L., Eickhoff C.R. (2015). A systematic review on the applications of resting-state fMRI in Parkinson's disease: Does dopamine replacement therapy play a role?. Cortex.

[bib71] Uchiyama M., Nishio Y., Yokoi K., Hirayama K., Imamura T., Shimomura T. (2012). Pareidolias: Complex visual illusions in dementia with Lewy bodies. Brain.

[bib72] Uddin L.Q., Kelly A.M., Biswal B.B., Castellanos F.X., Milham M.P. (2009). Functional connectivity of default mode network components: Correlation, anticorrelation, and causality. Human Brain Mapping.

[bib73] Vann Jones S.A., O'Brien J.T. (2014). The prevalence and incidence of dementia with Lewy bodies: A systematic review of population and clinical studies. Psychological Medicine.

[bib74] Vossel S., Geng J.J., Fink G.R. (2014). Dorsal and ventral attention systems: Distinct neural circuits but collaborative roles. The Neuroscientist: a Review Journal Bringing Neurobiology, Neurology and Psychiatry.

[bib75] Walker M.P., Ayre G.A., Cummings J.L., Wesnes K., McKeith I.G., O'Brien J.T. (2000). The clinician assessment of fluctuation and the one day fluctuation assessment scale: Two methods to assess fluctuating confusion in dementia. British Journal of Psychiatry.

[bib76] Wang K., Liang M., Wang L., Tian L., Zhang X., Li K. (2007). Altered functional connectivity in early Alzheimer's disease: A resting-state fMRI study. Human Brain Mapping.

[bib77] Wang L., Zang Y., He Y., Liang M., Zhang X., Tian L. (2006). Changes in hippocampal connectivity in the early stages of Alzheimer's disease: Evidence from resting state fMRI. NeuroImage.

[bib78] Weissman D.H., Roberts K.C., Visscher K.M., Woldorff M.G. (2006). The neural bases of momentary lapses in attention. Nature Neuroscience.

[bib79] Wen X., Yao L., Liu Y., Ding M. (2012). Causal interactions in attention networks predict behavioral performance. The Journal of Neuroscience: the Official Journal of the Society for Neuroscience.

