# Supplementary material for: Divergent functional connectivity during attentional processing in Lewy body dementia and Alzheimer's disease
Source: Cortex. 2017 Jul;92:8–18. doi: 10.1016/j.cortex.2017.02.016 (PMC5480774; doi:10.1016/j.cortex.2017.02.016)
Supplement: Supplementary file 1 [file mmc1.docx]

**Supplementary material**

1. **Supplementary material**

**Figure S1. Mean beta series estimates**. Activations for each condition and group (controls: red, AD : green, LBD: blue) as obtained by a GLM of the mean timeseries for each component. Significant contrasts between groups are marked with an asteriks. AD, AD; DMN, default mode network; EXEC, central executive network; DAN, dorsal attention network; HC, healthy controls; LBD, Lewy body dementia; VAN L, ventral attention network, left; VAN R, ventral attention network, right.


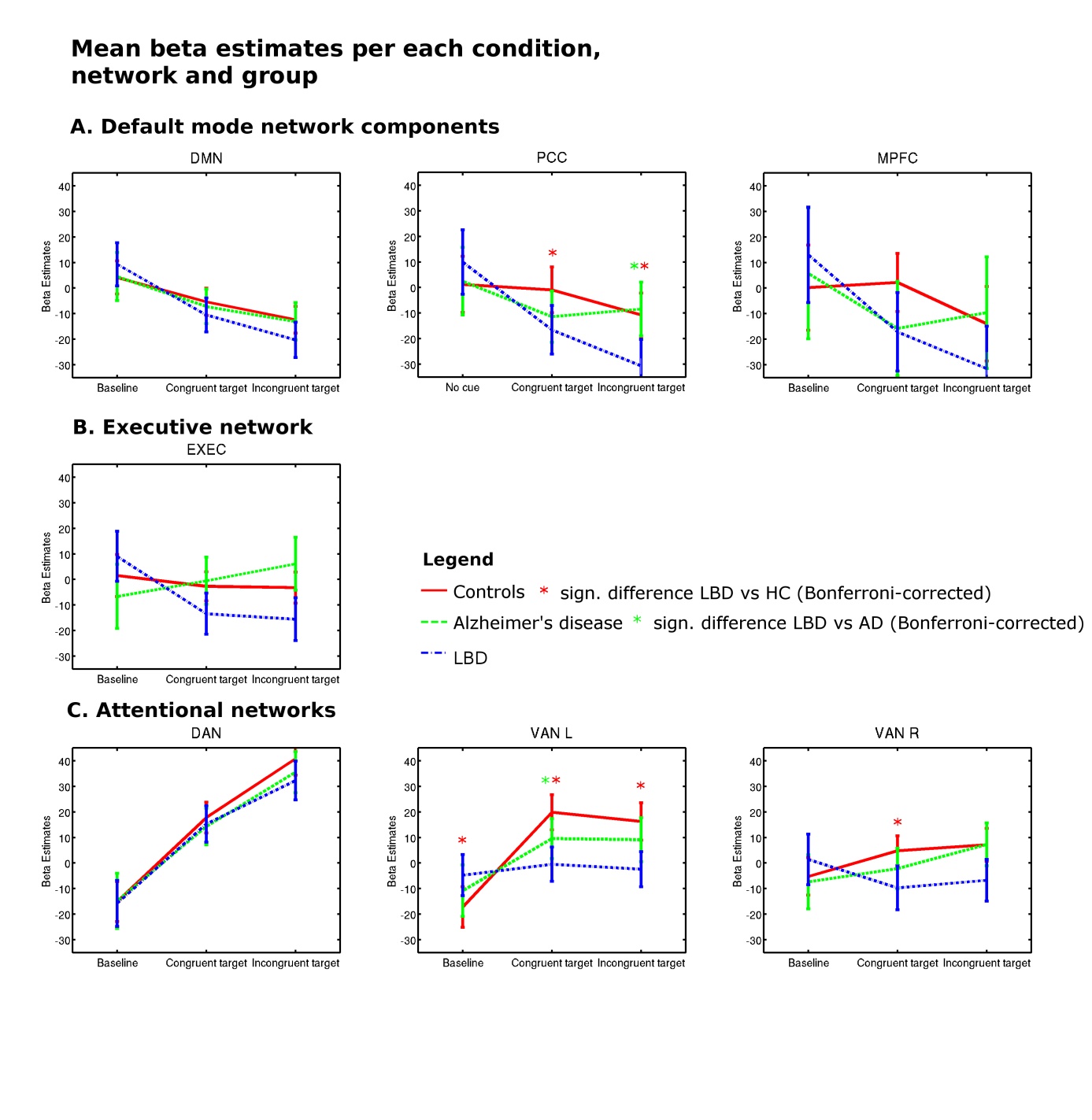


**Table S1. Z-normalised covariance coefficients for the baseline, congruent and incongruent condition.** All Z-scores above 2 are marked bold. DMN, default mode network; EXEC, central executive network; DAN, dorsal attention network; VAN_L, ventral attention network, left; VAN_R, ventral attention network, right.

|  | Controls | AD | LBD |
| --- | --- | --- | --- |
| Baseline (Z-score) |  |  |  |
| DMN-PCC | **9.13** | **8.451** | **9.948** |
| DMN-MPFC | 1.119 | -0.139 | 0.48 |
| DMN-EXEC | **3.084** | 1.788 | **2.327** |
| DMN_DAN | 1.951 | **3.741** | 1.885 |
| DMN-VAN_L | -0.217 | -0.362 | -0.1 |
| DMN-VAN_R | -1.66 | -1.512 | -1.301 |
| PCC-MPFC | **2.313** | 1.92 | 1.738 |
| PCC-EXEC | **3.413** | **2.894** | **2.996** |
| PCC-DAN | **2.249** | **4.632** | 1.897 |
| PCC-VAN_L | 1.196 | 1.34 | 0.885 |
| PCC-VAN_R | 0.174 | 0.239 | 0.002 |
| MPFC-EXEC | **2.216** | **2.126** | 1.784 |
| MPFC-DAN | 0.006 | 0.596 | -0.594 |
| MPFC-VAN_L | **2.388** | **2.074** | 1.497 |
| MPFC-VAN_R | **2.918** | **2.689** | 2.94 |
| EXEC-DAN | **4.161** | **3.683** | **3.177** |
| EXEC-VAN_L | 1.209 | 0.316 | 0.09 |
| EXEC-VAN_R | -0.045 | -0.195 | 0.283 |
| DAN-VAN_L | 1.727 | 0.688 | 0.164 |
| DAN-VAN_R | -0.08 | -0.172 | -0.55 |
| VAN_L-VAN_R | **2.475** | **2.532** | 1.379 |
| Congruent target (Z-score) | | | |
| DMN-PCC | **11.188** | **10.95** | **11.788** |
| DMN-MPFC | 1.124 | 0.299 | 0.124 |
| DMN-EXEC | **4.031** | **2.302** | **3.368** |
| DMN_DAN | **2.836** | **4.348** | **2.907** |
| DMN-VAN_L | 0.486 | 0.691 | -0.425 |
| DMN-VAN_R | -1.663 | -1.523 | -1.932 |
| PCC-MPFC | **2.702** | **2.354** | 1.721 |
| PCC-EXEC | **4.558** | **3.659** | **3.975** |
| PCC-DAN | **3.314** | **5.334** | **3.064** |
| PCC-VAN_L | 1.724 | **2.221** | 0.774 |
| PCC-VAN_R | 0.19 | 0.429 | -0.237 |
| MPFC-EXEC | **3.248** | **2.768** | **2.477** |
| MPFC-DAN | 0.648 | 0.968 | -0.568 |
| MPFC-VANL | **3.207** | **2.839** | 1.294 |
| MPFC-VAN_R | **4.188** | **4.742** | **4.088** |
| EXEC-DAN | **5.681** | **4.583** | **4.086** |
| EXEC-VANL | 1.44 | 0.226 | 0.257 |
| EXEC-VAN_R | 0.565 | 0.927 | 0.312 |
| DAN-VAN-L | **2.02** | 1.262 | 0.789 |
| DAN-VAN_R | 0.065 | 0.14 | -0.826 |
| VANL-VAN_R | **3.346** | **3.78** | 1.021 |
| Incongruent target (Z-score) | | | |
| DMN-PCC | **10.295** | **9.965** | **11.32** |
| DMN-MPFC | 0.894 | -0.219 | 0.054 |
| DMN-EXEC | **3.42** | **1.892** | **3.059** |
| DMN_DAN | **2.11** | **4.439** | **2.267** |
| DMN-VANL | -0.499 | 0.679 | -0.404 |
| DMN-VAN_R | -1.378 | -1.458 | -1.669 |
| PCC-MPFC | **2.57** | **2.265** | 1.71 |
| PCC-EXEC | **4.212** | **3.432** | **3.826** |
| PCC-DAN | **2.794** | **5.505** | **2.671** |
| PCC-VANL | 1.58 | **2.175** | 1.017 |
| PCC-VAN_R | 0.732 | 0.69 | 0.142 |
| MPFC-EXEC | **3.024** | **2.976** | **2.292** |
| MPFC-DAN | 0.04 | 0.745 | -0.703 |
| MPFC-VANL | **3.625** | **2.12** | 1.637 |
| MPFC-VAN_R | **3.629** | **3.581** | **3.903** |
| EXEC-DAN | **4.087** | **4.436** | **3.962** |
| EXEC-VANL | 1.645 | 0.467 | -0.183 |
| EXEC-VAN_R | 0.587 | 0.644 | 0.42 |
| DAN-VAN-L | **2.35** | 1.428 | 0.562 |
| DAN-VAN_R | 0.376 | -0.305 | -0.565 |
| VANL-VAN_R | **3.03** | **2.174** | 1.855 |

**Table S2. p-values of two-sided t-test between groups for all conditions.** Both uncorrected and FDR-corrected p-values are displayed. Significant values are marked bold. DMN, default mode network; EXEC, central executive network; DAN, dorsal attention network; VAN_L, ventral attention network, left; VAN_R, ventral attention network, right.

|  |  | Controls > AD | | AD >controls | | Controls > LBD | | LBD > Controls | | AD > LBD | | LBD > AD | |
| --- | --- | --- | --- | --- | --- | --- | --- | --- | --- | --- | --- | --- | --- |
|  |  | uncorrected | FDR-corrected | uncorrected | FDR-corrected | uncorrected | FDR-corrected | uncorrected | FDR-corrected | uncorrected | FDR-corrected | uncorrected | FDR-corrected |
| Baseline |  |  |  |  |  |  |  |  |  |  |  |  |  |
| DMN-PCC | 0.363 | | 0.705 | 0.637 | 0.841 | 0.878 | 0.878 | 0.122 | 0.993 | 0.936 | 0.936 | 0.064 | 1.000 |
| DMN-MPFC | 0.062 | | 0.625 | 0.938 | 0.938 | 0.196 | 0.469 | 0.804 | 0.993 | 0.797 | 0.837 | 0.203 | 1.000 |
| DMN-EXEC | 0.089 | | 0.625 | 0.911 | 0.938 | 0.178 | 0.469 | 0.822 | 0.993 | 0.710 | 0.829 | 0.290 | 1.000 |
| DMN_DAN | 0.985 | | 0.997 | **0.015** | 0.161 | 0.470 | 0.616 | 0.530 | 0.993 | **0.008** | 0.085 | 0.992 | 1.000 |
| DMN-VAN_L | 0.411 | | 0.705 | 0.589 | 0.841 | 0.571 | 0.666 | 0.429 | 0.993 | 0.663 | 0.819 | 0.337 | 1.000 |
| DMN-VAN_R | 0.537 | | 0.705 | 0.463 | 0.841 | 0.685 | 0.719 | 0.315 | 0.993 | 0.646 | 0.819 | 0.354 | 1.000 |
| PCC-MPFC | 0.321 | | 0.705 | 0.679 | 0.841 | 0.167 | 0.469 | 0.833 | 0.993 | 0.326 | 0.603 | 0.674 | 1.000 |
| PCC-EXEC | 0.319 | | 0.705 | 0.681 | 0.841 | 0.289 | 0.506 | 0.711 | 0.993 | 0.484 | 0.726 | 0.516 | 1.000 |
| PCC-DAN | 0.997 | | 0.997 | **0.003** | 0.063 | 0.340 | 0.509 | 0.660 | 0.993 | **<0.001** | **0.009** | 1.000 | 1.000 |
| PCC-VAN_L | 0.604 | | 0.705 | 0.396 | 0.841 | 0.334 | 0.509 | 0.666 | 0.993 | 0.240 | 0.601 | 0.760 | 1.000 |
| PCC-VAN_R | 0.537 | | 0.705 | 0.463 | 0.841 | 0.410 | 0.575 | 0.590 | 0.993 | 0.373 | 0.603 | 0.627 | 1.000 |
| MPFC-EXEC | 0.506 | | 0.705 | 0.494 | 0.841 | 0.260 | 0.496 | 0.740 | 0.993 | 0.257 | 0.601 | 0.743 | 1.000 |
| MPFC-DAN | 0.783 | | 0.866 | 0.217 | 0.841 | 0.201 | 0.469 | 0.799 | 0.993 | **0.048** | 0.254 | 0.952 | 1.000 |
| MPFC-VAN_L | 0.369 | | 0.705 | 0.631 | 0.841 | 0.066 | 0.320 | 0.934 | 0.993 | 0.129 | 0.541 | 0.871 | 1.000 |
| MPFC-VAN_R | 0.444 | | 0.705 | 0.556 | 0.841 | 0.517 | 0.639 | 0.483 | 0.993 | 0.577 | 0.808 | 0.423 | 1.000 |
| EXEC-DAN | 0.343 | | 0.705 | 0.657 | 0.841 | 0.076 | 0.320 | 0.924 | 0.993 | 0.164 | 0.552 | 0.836 | 1.000 |
| EXEC-VAN_L | 0.124 | | 0.652 | 0.876 | 0.938 | 0.054 | 0.320 | 0.946 | 0.993 | 0.365 | 0.603 | 0.635 | 1.000 |
| EXEC-VAN_R | 0.416 | | 0.705 | 0.584 | 0.841 | 0.685 | 0.719 | 0.315 | 0.993 | 0.759 | 0.837 | 0.241 | 1.000 |
| DAN-VAN_L | 0.067 | | 0.625 | 0.933 | 0.938 | **0.006** | 0.126 | 0.994 | 0.994 | 0.184 | 0.552 | 0.816 | 1.000 |
| DAN-VAN_R | 0.448 | | 0.705 | 0.552 | 0.841 | 0.248 | 0.496 | 0.752 | 0.993 | 0.299 | 0.603 | 0.701 | 1.000 |
| VAN_L-VAN_R | 0.594 | | 0.705 | 0.406 | 0.841 | 0.055 | 0.320 | 0.945 | 0.993 | **0.034** | 0.236 | 0.966 | 1.000 |
|  |  |  |  |  |  |  |  |  |  |  |  |  |  |
| Congruent target |  |  |  |  |  |  |  |  |  |  |  |  |  |
| DMN-PCC | 0.325 | | 0.682 | 0.675 | 0.956 | 0.284 | 0.345 | 0.716 | 0.999 | 0.472 | 0.533 | 0.528 | 1.000 |
| DMN-MPFC | 0.168 | | 0.589 | 0.832 | 0.956 | 0.100 | 0.209 | 0.900 | 0.999 | 0.409 | 0.505 | 0.591 | 1.000 |
| DMN-EXEC | **0.044** | | 0.577 | 0.956 | 0.956 | 0.154 | 0.230 | 0.846 | 0.999 | 0.804 | 0.804 | 0.196 | 1.000 |
| DMN_DAN | 0.936 | | 0.975 | 0.064 | 0.671 | 0.421 | 0.442 | 0.579 | 0.999 | **0.033** | 0.116 | 0.967 | 1.000 |
| DMN-VAN_L | 0.598 | | 0.797 | 0.402 | 0.886 | 0.117 | 0.223 | 0.883 | 0.999 | 0.075 | 0.226 | 0.925 | 1.000 |
| DMN-VAN_R | 0.572 | | 0.797 | 0.428 | 0.886 | 0.445 | 0.445 | 0.555 | 0.999 | 0.369 | 0.485 | 0.631 | 1.000 |
| PCC-MPFC | 0.290 | | 0.678 | 0.710 | 0.956 | **0.034** | 0.135 | 0.966 | 0.999 | 0.113 | 0.256 | 0.887 | 1.000 |
| PCC-EXEC | 0.146 | | 0.589 | 0.854 | 0.956 | 0.128 | 0.224 | 0.872 | 0.999 | 0.508 | 0.533 | 0.492 | 1.000 |
| PCC-DAN | 0.975 | | 0.975 | **0.025** | 0.519 | 0.287 | 0.345 | 0.713 | 0.999 | **0.004** | **0.043** | 0.996 | 1.000 |
| PCC-VAN_L | 0.714 | | 0.797 | 0.286 | 0.886 | 0.094 | 0.209 | 0.906 | 0.999 | **0.029** | 0.116 | 0.971 | 1.000 |
| PCC-VAN_R | 0.611 | | 0.797 | 0.389 | 0.886 | 0.296 | 0.345 | 0.704 | 0.999 | 0.202 | 0.347 | 0.798 | 1.000 |
| MPFC-EXEC | 0.261 | | 0.678 | 0.739 | 0.956 | 0.087 | 0.209 | 0.913 | 0.999 | 0.258 | 0.361 | 0.742 | 1.000 |
| MPFC-DAN | 0.667 | | 0.797 | 0.333 | 0.886 | **0.039** | 0.135 | 0.961 | 0.999 | **0.014** | 0.075 | 0.986 | 1.000 |
| MPFC-VAN_L | 0.289 | | 0.678 | 0.711 | 0.956 | **0.001** | **0.017** | 0.999 | 0.999 | **0.008** | 0.053 | 0.992 | 1.000 |
| MPFC-VAN_R | 0.721 | | 0.797 | 0.279 | 0.886 | 0.288 | 0.345 | 0.712 | 0.999 | 0.119 | 0.256 | 0.881 | 1.000 |
| EXEC-DAN | 0.082 | | 0.577 | 0.918 | 0.956 | **0.005** | **0.038** | 0.995 | 0.999 | 0.146 | 0.279 | 0.854 | 1.000 |
| EXEC-VAN_L | 0.070 | | 0.577 | 0.930 | 0.956 | 0.054 | 0.163 | 0.946 | 0.999 | 0.506 | 0.533 | 0.494 | 1.000 |
| EXEC-VAN_R | 0.656 | | 0.797 | 0.344 | 0.886 | 0.364 | 0.402 | 0.636 | 0.999 | 0.219 | 0.347 | 0.781 | 1.000 |
| DAN-VAN_L | 0.161 | | 0.589 | 0.839 | 0.956 | **0.035** | 0.135 | 0.965 | 0.999 | 0.231 | 0.347 | 0.769 | 1.000 |
| DAN-VAN_R | 0.536 | | 0.797 | 0.464 | 0.886 | 0.139 | 0.225 | 0.861 | 0.999 | 0.122 | 0.256 | 0.878 | 1.000 |
| VAN_L-VAN_R | 0.677 | | 0.797 | 0.323 | 0.886 | **0.002** | **0.017** | 0.998 | 0.999 | **<0.001** | **0.008** | 1.000 | 1.000 |
| Incongruent target |  |  |  |  |  |  |  |  |  |  |  |  |  |
| DMN-PCC | 0.474 | | 0.752 | 0.526 | 0.973 | 0.856 | 0.856 | 0.144 | 0.999 | 0.868 | 0.886 | 0.132 | 1.000 |
| DMN-MPFC | 0.129 | | 0.444 | 0.871 | 0.980 | 0.175 | 0.395 | 0.825 | 0.999 | 0.620 | 0.685 | 0.380 | 1.000 |
| DMN-EXEC | 0.057 | | 0.444 | 0.943 | 0.980 | 0.302 | 0.488 | 0.698 | 0.999 | 0.886 | 0.886 | 0.114 | 1.000 |
| DMN_DAN | 0.996 | | 0.999 | **0.004** | **0.042** | 0.549 | 0.618 | 0.451 | 0.999 | **0.003** | **0.031** | 0.997 | 1.000 |
| DMN-VAN_L | 0.936 | | 0.999 | 0.064 | 0.445 | 0.559 | 0.618 | 0.441 | 0.999 | 0.066 | 0.278 | 0.934 | 1.000 |
| DMN-VAN_R | 0.444 | | 0.752 | 0.556 | 0.973 | 0.374 | 0.504 | 0.626 | 0.999 | 0.435 | 0.571 | 0.565 | 1.000 |
| PCC-MPFC | 0.377 | | 0.752 | 0.623 | 0.980 | 0.099 | 0.347 | 0.901 | 0.999 | 0.175 | 0.355 | 0.825 | 1.000 |
| PCC-EXEC | 0.198 | | 0.521 | 0.802 | 0.980 | 0.256 | 0.448 | 0.744 | 0.999 | 0.607 | 0.685 | 0.393 | 1.000 |
| PCC-DAN | 0.999 | | 0.999 | **0.001** | **0.023** | 0.408 | 0.504 | 0.592 | 0.999 | **<0.001** | **0.005** | 1.000 | 1.000 |
| PCC-VAN_L | 0.801 | | 0.982 | 0.199 | 0.836 | 0.203 | 0.395 | 0.797 | 0.999 | **0.043** | 0.225 | 0.957 | 1.000 |
| PCC-VAN_R | 0.489 | | 0.752 | 0.511 | 0.973 | 0.207 | 0.395 | 0.793 | 0.999 | 0.219 | 0.383 | 0.781 | 1.000 |
| MPFC-EXEC | 0.517 | | 0.752 | 0.483 | 0.973 | 0.147 | 0.387 | 0.853 | 0.999 | 0.140 | 0.355 | 0.860 | 1.000 |
| MPFC-DAN | 0.841 | | 0.982 | 0.159 | 0.833 | 0.137 | 0.387 | 0.863 | 0.999 | **0.016** | 0.115 | 0.984 | 1.000 |
| MPFC-VAN_L | **0.020** | | 0.420 | 0.980 | 0.980 | **0.001** | **0.020** | 0.999 | 0.999 | 0.182 | 0.355 | 0.818 | 1.000 |
| MPFC-VAN_R | 0.526 | | 0.752 | 0.474 | 0.973 | 0.593 | 0.623 | 0.407 | 0.999 | 0.564 | 0.685 | 0.436 | 1.000 |
| EXEC-DAN | 0.714 | | 0.937 | 0.286 | 0.973 | 0.385 | 0.504 | 0.615 | 0.999 | 0.185 | 0.355 | 0.815 | 1.000 |
| EXEC-VAN_L | 0.073 | | 0.444 | 0.927 | 0.980 | **0.007** | **0.048** | 0.993 | 0.999 | 0.186 | 0.355 | 0.814 | 1.000 |
| EXEC-VAN_R | 0.537 | | 0.752 | 0.463 | 0.973 | 0.405 | 0.504 | 0.595 | 0.999 | 0.368 | 0.515 | 0.632 | 1.000 |
| DAN-VAN_L | 0.123 | | 0.444 | 0.877 | 0.980 | **0.005** | **0.048** | 0.995 | 0.999 | 0.095 | 0.334 | 0.905 | 1.000 |
| DAN-VAN_R | 0.148 | | 0.444 | 0.852 | 0.980 | 0.062 | 0.262 | 0.938 | 0.999 | 0.349 | 0.515 | 0.651 | 1.000 |
| VAN_L-VAN_R | 0.147 | | 0.444 | 0.853 | 0.980 | **0.040** | 0.208 | 0.960 | 0.999 | 0.271 | 0.437 | 0.729 | 1.000 |

**Table S3. Reaction times (RT) and error rates for all three groups**

|  | Controls |  | AD |  | LBD |  | AD vs LBD |
| --- | --- | --- | --- | --- | --- | --- | --- |
|  | Mean | S.D. | Mean | S.D. | Mean | S.D. | p-value |
| Congruent target: error rate (%) | 1.32 | 1.55 | 4.24 | 5.73 | 4.86 | 5.28 | 0.694 |
| Incongruent target: error rate (%) | 1.72 | 1.31 | 10.76 | 10.78 | 16.34 | 12.74 | 0.114 |
| Congruent target: RT (ms) | 898.33 | 108.32 | 1064.38 | 193.49 | 1296.61 | 250.21 | **0.001** |
| Congruent target: RT (ms) | 1233.91 | 235.27 | 1543.38 | 310.35 | 1850.03 | 419.07 | **0.007** |

**Supplementary results**

**Beta series activations**

Mean activations were obtained by calculating the mean beta value from the beta series correlation approach for each component, condition (baseline, congruent, incongruent) and group. The results are displayed in Figure S1 in the supplementary material.

In the DMN, there was no significant effect of group (p = 0.364), but a significant effect of condition (p < 0.001). The post-hoc t-tests revealed significant values for the contrasts between all conditions (all p-values < 0.001). With regard to the PCC, we found significant effects of group (p = 0.003), condition (p < 0.001) and group x condition interaction (p = 0.009). We found a significant differences between all conditions in LBD (all p-values < 0.001), but there were no significant difference between conditions in AD or controls (p ≥ 0.180). There was more deactivation of the PCC in LBD when compared to AD for the incongruent target (p = 0.027); and for both congruent (p = 0.036) and incongruent targets (p = 0.045) when compared to controls. The other between-groups differences did not reach significance (p ≥ 0.621). In the MPFC there was neither a significant effect of group (p = 0.367) nor of condition (p = 0.068). The same held true for the EXEC (effect of group: p = 0.068; effect of condition: p = 0.246). In the DAN, there was no effect of group (p = 0.194), but a significant effect of condition (p < 0.001). Post-hoc t-tests revealed significant differences between all conditions (all p < 0.001) with increasing activation from baseline to congruent target to incongruent target. In the left ventral attention network (VAN), there was a significant effect of group (p < 0.001), condition (p < 0.001,) and interaction of group x condition (p < 0.001). In controls, there was a significant increase in activation from baseline to congruent in the left VAN as well as from baseline to incongruent states (both p < 0.001). In AD, only the increase from baseline to congruent reached a significant level (p = 0.009). All other differences were not significant (p ≥ 0.252). A between-group analysis showed more activation for the congruent target in AD compared to LBD (p = 0.045) in the left VAN. All conditions yielded a significant difference between controls and LBD (baseline: p = 0.027; congruent: p < 0.001; incongruent: p < 0.001), showing less activation during baseline but more activation during the other conditions in controls. All other differences between groups were not significant (p-values ≥ 0.072). With regard to the right VAN, we observed a significant effect of group (p = 0.011), but not of condition (p = 0.200). The interaction group x condition was significant (p = 0.028) with a post-hoc t-test demonstrating a higher activation in controls compared to LBD (p = 0.036). The other post-hoc comparisons did not reach a significant level (p ≥ 0.216).

**Comparison of movement parameters between groups**

**Translation parameters**

ANOVA between groups (HC, AD, LBD)

p = 0.475

Figure S2: Means and standard deviations of translation parameters (in mm)

**Rotational movement**

ANOVA between groups (HC,AD, LBD)

p = 0.042

Post-hoc T-tests (Bonferroni-corrected)

AD vs LBD p = 1.000

AD vs HC p = 0.045

LBD vs HC = 0.194

Figure S3:

Means and standard deviations of rotational parameters (in radians)

**Overlap of functional connectivity of the present study with known cortical networks as in Smith et al.**

We performed a quantitative analysis of the percentage overlap between exemplary resting state networks from the analysis of Smith et. al (2009) and those derived in our analysis. To do so, we binarized both networks at 70% voxel threshold and then calculated the percentage of the overlap. We found a good overlap between the executive networks and the default mode networks. Concerning the attention networks, calculating the overlap was challenging. As our ICA resulted in 37 components and split up the networks into the dorsal and ventral attention networks, whereas Smith et al.’s analysis mainly yielded 10 independent components, of which two were identified as frontoparietal networks, therefore our attentional networks did not correspond exactly to the attentional networks as in Smith et al. Although Smith et al. had an analysis that included 20 and 70 components, these were not exactly assigned in the publication and therefore we did not use them for comparison. Furthermore, the networks in Smith et al.’s publication were based on resting state data and thus may have some differences to the networks originating from our task-based study. Concerning the attentional networks, we found the most overlap between Smith et al.’s right frontoparietal network and the right ventral attentional network in our study and Smith et al. left frontoparietal network and the dorsal attention network from our ICA.

Figure S4: Overlap of functional connectivity of the present study with exemplary resting state networks as in Smith et al. EXEC: executive network, DAN: dorsal attention network, L VAN: left ventral attention network, R VAN: right attention network, DMN: default mode network, R FPN: right frontoparietal network, L FPN: left frontoparietal network.
